# Completion Probabilities and Parallel Restart Strategies under an Imposed Deadline

**DOI:** 10.1371/journal.pone.0164605

**Published:** 2016-10-12

**Authors:** Jan-Hendrik Lorenz

**Affiliations:** Institut für Theoretische Informatik, Universität Ulm, 89069 Ulm, Germany; Nankai University, CHINA

## Abstract

Let *A* be any fixed cut-off restart algorithm running in parallel on multiple processors. If the algorithm is only allowed to run for up to time *D*, then it is no longer guaranteed that a result can be found. In this case, the probability of finding a solution within the time *D* becomes a measure for the quality of the algorithm. In this paper we address this issue and provide upper and lower bounds for the probability of *A* finding a solution before a deadline passes under varying assumptions. We also show that the optimal restart times for a fixed cut-off algorithm running in parallel is identical for the optimal restart times for the algorithm running on a single processor. Finally, we conclude that the odds of finding a solution scale superlinearly in the number of processors.

## 1 Introduction

Restart strategies are commonly used in probabilistic algorithms. If the current computation takes too long, then the algorithm is started over with a different random seed. Deciding when to restart is an important task in designing an algorithm and several strategies are known. Luby et al. introduced the fixed cut-off strategy (*t*, *t*, …) in [1]. This means after *t* steps the algorithm is restarted. They also showed that this strategy is optimal for a certain value of *t*. The expected runtime is $E\left[A_{S}^{t}\right]=\frac{1}{F(t)}\left(t-\sum_{t^{\prime }<t}F\left(t^{\prime }\right)\right)$, where $A_{S}^{t}$ is a random variable describing the runtime of a fixed cut-off strategy using restart time *t* and *F* is the cumulative runtime distribution. However, for deciding the value of *t*, the exact runtime distribution has to be known. In most real applications this is difficult to achieve. Luby et al. also introduced Luby’s universal strategy in [1], which is a strategy that does not require any knowledge about the runtime distribution. And they showed that, compared to the optimal strategy, Luby’s universal strategy is only worse by a logarithmic factor.

One interesting question in the context of restart strategies is whether restarts are helpful at all, and if so, under which conditions. This question has been answered by van Moorsel and Wolter in [2]: They showed that restarts at time *t* are useful if *E*[*A*] < *E*[*A* − *t* ∣ *A* > *t*] holds where *A* is a random variable describing the runtime of the respective algorithm. This behaviour is shown by heavy-tailed distributions. However, in general such a condition cannot always be analysed since the expected runtime may be infinite.

Restart strategies are a way to acquire a solution for a hard problem in (on average) less time. Another possibility is to increase the available computational power. Since some years ago even personal computer usually possess more than one processor which makes parallel processing a valid option. Of course, restart algorithms can also be operated on several processors at once. Luby and Ertel noted in [3] that a fixed cut-off strategy is not necessarily optimal in the case of parallel computing. And in 2011 Shylo et al. showed in [4] that the optimal restart value (regarding expected runtime) for a single process fixed cut-off strategy does not necessarily coincide with the optimal value in the parallel case. Under certain conditions they showed that the optimal restart time in the parallel case is greater than the restart time in the single process case. They also showed that the speedup-ratio is sublinear. While in 2014 Cire et al. showed in [5] that the speedup-ratio of Luby’s universal strategy scales asymptotically linearly with the number of processors.

Closely related to the idea of restarts is a concept called portfolio. The basic idea of a portfolio is to run different algorithms solving the same problem. They can either run in parallel on multiple processors or share a single processor. An introduction to portfolio theory can be found in [6]. Shylo at al. examined the relationship between restart strategies and portfolios in [7]. They found that the speedup ratio of a restarted algorithm running on *n* processors and two restarted algorithms running on (in total) *n* processors is bounded by a small constant.

In several real-life scenarios algorithms are not allowed to run for an arbitrarily long time, instead the run is aborted after a fixed time *D*. This is commonly denoted as a deadline. Under these circumstances it cannot be guaranteed that a solution will be found. Therefore the probability of finding a solution within the deadline is, in some cases, a more interesting issue than the expected runtime (which might not even exist). Van Moorsel and Wolter showed in [8] that a local extremum regarding probability can be found at the so-called equi-hazard intervals. This means, if *t*_1_, *t*_2_ … *t*_*k*_ are the restart times, then they are a local extremum iff

$$\frac{f(t_{1})}{1-F(t_{1})}=\frac{f(t_{2})}{1-F(t_{2})}=\cdots =\frac{f(t_{k})}{1-F(t_{k})}.$$

Here *F* is the runtime distribution and *f* is its derivate, the density function.

**Our contribution:** We consider restarted algorithms running in parallel on several processors. The used restarted algorithms are using the fixed cut-off strategy. We are imposing a deadline on such algorithms and analyse the probability of finding a solution within the deadline (Theorem 3.2). We then compare the probabilities of finding a solution on a single processor with finding a solution on multiple processors. We provide upper and lower bounds on the probabilities of multiple processors finding a solution under varying assumptions regarding both the restart times and the deadline. The respective bounds are given in Theorem 3.4 and Theorem 3.5. We also show that the optimal restart time regarding completion probability in the case of a fixed cut-off algorithm running on a single processor is also optimal for the algorithm running in parallel on multiple processors. This result is summarised in Theorem 4.2.

## 2 Notation

In this work we consider randomised algorithms and use random variables to describe the runtime of such algorithms. We write *Pr*(*X*) to describe the probability of an event *X*. Let A be a randomised algorithm and *A* be a random variable describing its runtime on the respective input. The cumulative runtime distribution is given by *F*(*t*) = *Pr*(*A* ≤ *t*) for t∈R, i.e. the probability that A finds a solution within time *t*. We use *f* as symbol to describe the probability density function $f\left(t\right)=\frac{d}{dt}F\left(t\right)$. Other than their existence we do not use further assumptions about the cumulative runtime distribution and the probability density function.

We only consider the fixed cut-off strategy in this work which means that whenever talking about an algorithm or a process, we implicitly mean an algorithm using the fixed cut-off strategy. The fixed cut-off strategy was defined by Luby et. al in [1].

**Strategy 2.1** ([1]). *Let* A *be a randomised algorithm and let* t∈R *be any time with t* > 0. *We slightly modify the behaviour of* A *to obtain a new algorithm called* $A_{t}$. *First add a timer T to* A *which measures the elapsed time*. *Whenever T exceeds t and* A *did not find a solution, reset the time* *T* *to zero and restart* A *(with new independent random choices)*. *Repeat this behaviour until a solution is found*.

We also define the use of deadlines.

**Definition 2.2**. *Let* A be an algorithm and D∈R *be a time*, *here called deadline*. *We modify the behaviour of* A *to obtain an algorithm* $A_{D}$ *which uses a deadline*. *First add a timer T which measures the elapsed time*. *If T exceeds D*, *then the computation of* A *is aborted even if the computation is not complete*.

## 3 Completion Probabilities and Parallel Restart Strategies

The probability of a single process finishing within a given time frame was calculated by Wu:

**Theorem 3.1** ([9]). *Let D be the deadline and t be the restart time*, *also let* *A*_*t*_ *be a random variable describing the runtime of an algorithm* A *with restart time* *t*. *Then the number of restarts is* $k=\lfloor \frac{D}{t}\rfloor$, *and the leftover time is* *t*_0_ = *D* − *k* ⋅ *t*. *The probability that algorithm* A *running on a single processor finds a solution within the deadline is:*

$$Pr\left(A_{t}\leq D\right)=1-{\left(1-F\left(t\right)\right)}^{k}+{\left(1-F\left(t\right)\right)}^{k}F\left(t_{0}\right).$$ (1)

*The expected runtime (conditioned that the deadline is met) of algorithm A is:*

$$E\left[A_{t}|A_{t}\leq D\right]=E\left[A_{S}^{t}\right]\left(1-{\left(1-F\left(t\right)\right)}^{k}\right)+{\left(1-F\left(t\right)\right)}^{k}\left(t_{0}-\sum_{t^{\prime }=1}^{t_{0}-1}F\left(t^{\prime }\right)\right).$$ (2)

*Here* $E[A_{S}^{t}]$ *is the expected runtime of the fixed cut-off strategy without any deadline*.

This probability can be equivalently stated as *Pr*(*A*_*t*_ ≤ *D*) = 1 − (1 − *F*(*t*))^*k*^(1 − *F*(*t*_0_)). These calculations can be easily adapted to the case of parallel restart strategies.

**Theorem 3.2**. *Let D be the deadline*, *n* *the number of processors and* *t*_*n*_ *the restart time for* *n* *processors*, *also let* $A_{t_{n}}^{n}$ *be a random variable describing the runtime of an algorithm* A *running on* *n* *processors and using restart time* *t*_*n*_. *Then the number of restarts is* $k_{n}=\left\lfloor \frac{D}{t_{n}}\right\rfloor$ *and the leftover time is* *t*_*n*,0_ = *D* − *k*_*n*_ ⋅ *t*_*n*_. *The probability that algorithm* A *finds a solution within the deadline is:*

$$Pr\left(A_{t_{n}}^{n}\leq D\right)=1-{\left(1-F\left(t_{n}\right)\right)}^{k_{n}\cdot n}+{\left(1-F\left(t_{n}\right)\right)}^{n\cdot k_{n}}\left(1-{\left(1-F\left(t_{n,0}\right)\right)}^{n}\right).$$ (3)

*The expected runtime of algorithm A (conditioned that the deadline is met) can be upper bounded by*

$$E\left[A_{t_{n}}^{n}|A_{t_{n}}^{n}\leq D\right]\leq t_{n}\frac{1-{\left(1-F\left(t_{n}\right)\right)}^{n\cdot k_{n}}}{1-{\left(1-F\left(t_{n}\right)\right)}^{n}}+{\left(1-F\left(t_{n}\right)\right)}^{n\cdot k_{n}}t_{n,0}.$$ (4)

*Proof*. First we show the claimed probability. Clearly (1 − *F*(*t*_*n*_))^*k*_*n*_ ⋅ *n*^ describes the probability that none of the processors finds a solution within their respective *k*_*n*_ restarts under restart time *t*_*n*_. Therefore, (1 − (1 − *F*(*t*_*n*_))^*k*_*n*_ ⋅ *n*^) is the probability that at least one process finds a solution within the given time. Following this argument, (1 − (1 − *F*(*t*_*n*,0_))^*n*^) is the probability that at least one of the processes finds a solution within the leftover time.

To evaluate the expected runtime of the restart algorithm, we start by constructing an algorithm *B* behaving similarly to A. Let $B_{t_{n}}^{n}$ be a random variable describing the runtime of *B*. The algorithm *B* can only return a solution after *l* ⋅ *t*_*n*_ steps where 1 ≤ *l* ≤ *k*_*n*_, l∈N, or after *k*_*n*_ ⋅ *t*_*n*_ + *t*_*n*,0_ steps. In other words, if one of the processors finds a solution, the algorithm *B* waits until it is supposed to restart and then returns its solution. Obviously, *B* performs worse than the original algorithm. Therefore, we have:

$$E\left[A_{t_{n}}^{n}|A_{t_{n}}^{n}\leq D\right]\leq E\left[B_{t_{n}}^{n}|B_{t_{n}}^{n}\leq D\right].$$

Now we analyse the expected runtime of *B*. $E[B_{t_{n}}^{n}|B_{t_{n}}^{n}\leq D]$ is given by:

$$\begin{array}{ll} t_{n}\cdot \left(1-{\left(1-F(t_{n})\right)}^{n}\right)+{\left(1-F(t_{n})\right)}^{n}\left(t_{n}+E[B_{t_{n}}^{n}|B_{t_{n}}^{n}\leq D-t_{n}]\right) & \text{if }D\geq t_{n}\\ t_{n,0} & \text{if }D<t_{n}. \end{array}$$

The first part *t*_*n*_ ⋅ (1 − (1 − *F*(*t*_*n*_))^*n*^) describes the probability that a solution is found within the restart time, in this case $B_{t_{n}}^{n}$ runs for exactly *t*_*n*_ steps. Otherwise it requires $\left(t_{n}+E\left[B_{t_{n}}^{n}|B_{t_{n}}^{n}\leq D-t_{n}\right]\right)$ steps. For *D* ≥ *t*_*n*_ this can be simplified to:

$$\begin{array}{ll} & t_{n}\cdot (1-{\left(1-F(t_{n})\right)}^{n})+{\left(1-F(t_{n})\right)}^{n}(t_{n}+E[B_{t_{n}}^{n}|B_{t_{n}}^{n}\leq D-t_{n}])\\ = & t_{n}-t_{n}{\left(1-F(t_{n})\right)}^{n}+t_{n}{\left(1-F(t_{n})\right)}^{n}+{\left(1-F(t_{n})\right)}^{n}E[B_{t_{n}}^{n}|B_{t_{n}}^{n}\leq D-t_{n}]\\ = & t_{n}+{\left(1-F(t_{n})\right)}^{n}E[B_{t_{n}}^{n}|B_{t_{n}}^{n}\leq D-t_{n}]. \end{array}$$

Unrolling this equation until *D* < *t*_*n*_ yields the following:

$$\begin{array}{ll} & t_{n}+{\left(1-F(t_{n})\right)}^{n}E[B_{t_{n}}^{n}|B_{t_{n}}^{n}\leq D-t_{n}]\\ = & t_{n}\cdot \underset{0\leq l\leq k_{n}-1}{\sum^{\text{​}}}{\left(1-F(t_{n})\right)}^{n\cdot l}+{\left(1-F(t_{n})\right)}^{n\cdot k_{n}}t_{n,0} \end{array}$$

Using the geometric series, we obtain that the expected value is:

$$E\left[B_{t_{n}}^{n}|B_{t_{n}}^{n}\leq D\right]=t_{n}\cdot \frac{1-{\left(1-F\left(t_{n}\right)\right)}^{n\cdot k_{n}}}{1-{\left(1-F\left(t_{n}\right)\right)}^{n}}+{\left(1-F\left(t_{n}\right)\right)}^{n\cdot k_{n}}t_{n,0}$$

This concludes the proof since $E\left[A_{t_{n}}^{n}|A_{t_{n}}^{n}\leq D\right]\leq E\left[B_{t_{n}}^{n}|B_{t_{n}}^{n}\leq D\right]$.

Again, the probability can be expressed equivalently by $Pr\left(A_{t_{n}}^{n}\leq D\right)=1-{\left(1-F\left(t_{n}\right)\right)}^{k_{n}\cdot n}{\left(1-F\left(t_{n,0}\right)\right)}^{n}$. This notation is used in some of the proofs. In the special case of *t* = *t*_*n*_ and *t*_0_ = 0 this leads to a first result.

**Corollary 3.3**. *If* *t* = *t*_*n*_ *and* *t*_0_ = 0, *then the following equation holds*.

$$Pr\left(A_{t_{n}}^{n}\leq D\right)=Pr\left(A_{t}\leq n\cdot D\right).$$ (5)

*Proof*. If *t*_0_ = 0, then $k=n\cdot \frac{D}{t}=n\cdot k_{n}$, the rest follows from inserting this into the formulas.

In other words, a single process needs deadlines being *n* times greater to achieve the same probability of finding a solution, as compared to a process running in parallel on *n* processors. In the following, we analyse the probability of both, the parallel strategy and the single strategy, in the case when the deadline is fixed. We start by using some restrictions on the allowed strategies. Later on strategies with relaxed restrictions are analysed. In the first case we consider identical restart times for both strategies and no leftover time.

**Theorem 3.4**. *Let D be a deadline and* $n=2^{i},i\in N$, *be the number of processors*. *Let t be a restart time and define* *A*_*t*_ *to be a random variable describing the runtime of the single strategy using restart time t*. *Define* $A_{t}^{n}$ *analogously for the parallel restart strategy running on n processors*. *Then the following holds:*

$$Pr\left(A_{t}\leq D\right)\cdot \prod_{0\leq j<i}\left(1+{\left(1-F\left(t\right)\right)}^{2^{j}\cdot k}\right){\left(1-F\left(t_{0}\right)\right)}^{2^{j}}=Pr\left(A_{t}^{n}\leq D\right).$$ (6)

*Proof*. This can be shown by using an inductive argument. First consider the case *n* = 2^1^ and analyse $\frac{Pr(A_{t}\leq D)}{Pr(A_{t}^{2}\leq D)}$.

$$\frac{Pr(A_{t}\leq D)}{Pr(A_{t}^{2}\leq D)}=\frac{1-{\left(1-F\left(t\right)\right)}^{k}\left(1-F\left(t_{0}\right)\right)}{1-{\left(1-F\left(t\right)\right)}^{k\cdot 2}{\left(1-F\left(t_{0}\right)\right)}^{2}}=\frac{1}{1+{\left(1-F\left(t\right)\right)}^{k}\left(1-F\left(t_{0}\right)\right)}$$

Therefore $Pr\left(A_{t}\leq D\right)\cdot \left(1+{\left(1-F\left(t\right)\right)}^{k}\left(1-F\left(t_{0}\right)\right)\right)=Pr\left(A_{t}^{2}\leq D\right)$ holds. Now we examine the case of *n* = 2^*i*^ for *i* > 1.

$$\begin{array}{l} \frac{Pr(A_{t}^{n/2}\leq D)}{Pr(A_{t}^{n}\leq D)}=\frac{1-{\left(1-F(t)\right)}^{k\cdot 2^{i-1}}{\left(1-F(t_{0})\right)}^{2^{i-1}}}{1-{\left(1-F(t)\right)}^{k\cdot 2^{i}}{\left(1-F(t_{0})\right)}^{2^{i}}}\\ =\frac{1-{\left(1-F(t)\right)}^{k\cdot 2^{i-1}}{\left(1-F(t_{0})\right)}^{2^{i-1}}}{\left(1-{\left(1-F(t)\right)}^{k\cdot 2^{i-1}}{\left(1-F(t_{0})\right)}^{2^{i-1}})(1+{\left(1-F(t)\right)}^{k\cdot 2^{i-1}}{\left(1-F(t_{0})\right)}^{2^{i-1}}\right)}\\ =\frac{1}{1+{\left(1-F(t)\right)}^{k\cdot 2^{i-1}}{\left(1-F(t_{0})\right)}^{2^{i-1}}} \end{array}$$

Therefore, $Pr\left(A_{t}^{n/2}\leq D\right)\cdot \left(1+{\left(1-F\left(t\right)\right)}^{k\cdot 2^{i-1}}{\left(1-F\left(t_{0}\right)\right)}^{2^{i-1}}\right)=Pr\left(A_{t}^{n}\leq D\right)$ holds. By using the induction hypothesis the desired result follows.

We now analyse the case when restart times are not identical. We assume, however, that for an increasing number of processors the restart times are non-decreasing. Later on, we discuss whether this restriction is reasonable.

**Theorem 3.5**. *Let D be a deadline and* $n_{i}=2^{i}\cdot {k_{0}}^{i}$ *be the number of available processors*. *Let* *t*_*i*_ *be the restart time for the algorithm running on* *n*_*i*_ *processors and let* *t*_*i*_ ≤ *D* *and* ∀*i*: *t*_*i*+1_ ≥ *t*_*i*_. *Define* *k*_*i*_ *as the number of restarts of the algorithm running on* *n*_*i*_ *processors with restart time* *t*_*i*_, *where we require* ∀*i*: *k*_*i*_ ≥ 2. *Define* $A_{t_{i}}^{n_{i}}$ *as a random variable describing the runtime of the algorithm running on* *n*_*i*_ *processors with restart time* *t*_*i*_. *Define* *m* = *i* + *i*⌈log_2_ *k*_0_⌉. *Then*

$$Pr\left(A_{t_{0}}\leq D\right)\cdot \prod_{1\leq j\leq i}\left(1+{\left(1-F\left(t_{j}\right)\right)}^{2^{j}k_{0}^{j}}\right)\leq Pr\left(A_{t_{i}}^{n_{i}}\leq D\right)$$ (7)

*and*

$$Pr\left(A_{t_{i}}^{n_{i}}\leq D\right)\leq Pr\left(A_{t_{i}}\leq D\right)\cdot \prod_{0\leq j<m}\left(1-{\left(1-F\left(t_{i}\right)\right)}^{2^{j}k_{i}}\left(1-F\left(t_{i,0}\right)\right)\right))$$ (8)

*holds*.

*Proof*. We start by showing a lower bound as in inequality Eq 7. This can be shown by induction. First examine the probabilities for the case of *n*_0_ and *n*_1_.

$$\frac{Pr(A_{t_{0}}^{n_{0}}\leq D)}{Pr(A_{t_{1}}^{n_{1}}\leq D)}=\frac{1-{\left(1-F\left(t_{0}\right)\right)}^{k_{0}}+{\left(1-F\left(t_{0}\right)\right)}^{k_{0}}\cdot F\left(t_{0,0}\right)}{1-{\left(1-F\left(t_{1}\right)\right)}^{2k_{0}\cdot k_{1}}+{\left(1-F\left(t_{1}\right)\right)}^{2k_{0}\cdot k_{1}}\cdot \left(1-{\left(1-F\left(t_{1,0}\right)\right)}^{2k_{0}}\right)}$$

Since *t*_1_ ≥ *t*_0_ the value of this expression is **less or equal** to:

$$\frac{1-{\left(1-F\left(t_{0}\right)\right)}^{k_{0}+1}}{1-{\left(1-F\left(t_{0}\right)\right)}^{2k_{0}\cdot k_{1}}}\overset{k_{1}\geq 2}{\leq }\frac{1-{\left(1-F\left(t_{0}\right)\right)}^{2k_{0}}}{1-{\left(1-F\left(t_{0}\right)\right)}^{4k_{0}}}=\frac{1}{1+{\left(1-F\left(t_{0}\right)\right)}^{2k_{0}}}$$

Therefore $Pr\left(A_{t_{0}}^{n_{0}}\leq D\right)\cdot \left(1+{\left(1-F\left(t_{1}\right)\right)}^{2k_{0}}\right)\leq Pr\left(A_{t_{1}}^{n_{1}}\leq D\right)$ holds. At this point we analyse the inductive step $Pr\left(A_{t_{i}}^{n_{i}}\leq D\right)/Pr\left(A_{t_{i+1}}^{n_{i+1}}\leq D\right)$. We start by examining the numerator.

$$\begin{array}{ll} Pr(A_{t_{i}}^{n_{i}}\leq D) & =1-{\left(1-F(t_{i})\right)}^{2^{i}\cdot k_{0}^{i}\cdot k_{i}}\cdot {\left(1-F(t_{i,0})\right)}^{2^{i}k_{0}^{i}}\\ & \leq 1-{\left(1-F(t_{i})\right)}^{2^{i}\cdot k_{0}^{i}\cdot k_{i}}\cdot {\left(1-F(t_{i})\right)}^{2^{i}k_{0}^{i}}\\ & =1-{\left(1-F(t_{i})\right)}^{2^{i}\cdot k_{0}^{i}\cdot (k_{i}+1)} \end{array}$$

The first step follows because the leftover time *t*_*i*,0_ is less or equal to the restart time *t*_*i*_. Therefore *F*(*t*_*i*,0_) ≤ *F*(*t*_*i*_). Since the restart times are non-decreasing the number of restarts are non-increasing, i.e., *k*_0_ ≥ *k*_*i*_. By using this fact we obtain $Pr\left(A_{t_{i}}^{n_{i}}\leq D\right)\leq 1-{\left(1-F\left(t_{i}\right)\right)}^{2^{i+1}\cdot k_{0}^{i+1}}$.

Now we examine the denominator $Pr(A_{t_{i+1}}^{n_{i+1}}\leq D)$.

$$\begin{array}{l} 1-{\left(1-F(t_{i+1})\right)}^{2^{i+1}\cdot k_{0}^{i+1}\cdot k_{i+1}}+{\left(1-F(t_{i+1})\right)}^{2^{i+1}\cdot k_{0}^{i+1}\cdot k_{i+1}}\cdot (1-{\left(1-F(t_{i+1,0})\right)}^{2^{i+1}k_{0}^{i+1}})\\ \geq 1-{\left(1-F(t_{i+1})\right)}^{2^{i+1}\cdot k_{0}^{i+1}\cdot k_{i+1}}\geq 1-{\left(1-F(t_{i})\right)}^{2^{i+1}\cdot k_{0}^{i+1}\cdot k_{i+1}} \end{array}$$

The last step follows from the fact that *F*(*t*_*i*_) ≤ *F*(*t*_*i*+1_). By using the requirement that there are at least two restarts, we obtain $Pr\left(A_{t_{i+1}}^{n_{i+1}}\leq D\right)\geq 1-{\left(1-F\left(t_{i}\right)\right)}^{2^{i+2}\cdot k_{0}^{i+1}}$. With those two observations the quotient $Pr\left(A_{t_{i}}^{n_{i}}\leq D\right)/Pr\left(A_{t_{i+1}}^{n_{i+1}}\leq D\right)$ can now be analysed.

$$\begin{array}{ll} & \frac{Pr(A_{t_{i}}^{n_{i}}\leq D)}{Pr(A_{t_{i+1}}^{n_{i+1}}\leq D)}\\ \leq & \frac{1-{\left(1-F(t_{i})\right)}^{2^{i+1}k_{0}^{i+1}}}{1-{\left(1-F(t_{i})\right)}^{2^{i+2}k_{0}^{i+1}}}\\ = & \frac{1}{1+{\left(1-F(t_{i})\right)}^{2^{i+1}k_{0}^{i+1}}} \end{array}$$

Therefore we can conclude that

$$Pr\left(A_{t}\leq D\right)\cdot \prod_{1\leq j\leq i}\left(1+{\left(1-F\left(t_{j}\right)\right)}^{2^{j}k_{0}^{j}}\right)\leq Pr\left(A_{t_{i}}^{n_{i}}\leq D\right).$$

We now move on to inequality Eq 8. First notice that $n_{i}=2^{i}k_{0}^{i}=2^{i+i\log_{2}k_{0}}\leq 2^{i+i\lceil \log_{2}k_{0}\rceil }$. Define *n*′ = 2^*i*+*i*⌈log_2_*k*_0_⌉^, then it is clear that $Pr\left(A_{t_{i}}^{n_{i}}\leq D\right)\leq Pr\left(A_{t_{i}}^{n^{\prime }}\leq D\right)$ holds. The rest follows from Theorem 3.4.

## 4 Optimal Restart Time

For the proof of Theorem 3.5 we used the assumption that the restart times are non-decreasing for an increasing number of processors. We want to assess this assumption and evaluate under which conditions it is reasonable. Before analysing the optimal restart time, we should first point out that there are multiple definitions for the restart time being optimal. On the one hand, the restart time can be chosen such that the expected runtime is minimised, and on the other hand, the restart time can be chosen such that the completion probability is maximised. The optimal values do not have to be the same. In Lemma 4.1 a condition for the optimal restart time regarding the expected runtime is analysed.

Let *t*′ be the optimal restart time (regarding the expected runtime) for an algorithm operating on one processor and let $t_{n}^{\prime }$ be the respective optimal restart time for the algorithm operating on *n* processors. Define *T*(*t*′) as the expected runtime for the algorithm running on one processor and let $T(t_{n}^{\prime })$ be the respective runtime on *n* processors. Shylo et al. showed in Theorem 2 of [4] that $\frac{T(t^{\prime })}{T(t_{n}^{\prime })}\leq n$ always holds.

They also showed that if the hazard function $h\left(x\right)=\frac{f(x)}{1-F(x)}$ is unimodal and $\frac{T(t^{\prime })}{T(t_{n}^{\prime })}<n$, then $t^{\prime }<t_{n}^{\prime }$ holds for all *n*. While the following Lemma is not stated explicitly in their work, the result is very similar to Theorem 3 in their work. All techniques used in our proof are from their work, the following Lemma is therefore implicit in their work.

**Lemma 4.1**. *If the hazard function of the runtime distribution is unimodal and* $\frac{T(t_{m}^{\prime })}{T(t_{n}^{\prime })}<\frac{n}{m}$ *for* *n* > *m*, *then* $t_{n}^{\prime }>t_{m}^{\prime }$.

*Proof*. Shylo et al. showed in Corollary 2 of [4] that the expected runtime of an algorithm running on *n* processors and using the optimal restart time $t_{n}^{\prime }$ is $T\left(t_{n}^{\prime }\right)=\frac{1-F(t_{n}^{\prime })}{n\cdot f(t_{n}^{\prime })}$. Using this we have:

$$\begin{array}{ll} & \frac{1-F(t_{m}^{\prime })}{m\cdot f(t_{m}^{\prime })}\frac{n\cdot f(t_{n}^{\prime })}{1-F(t_{n}^{\prime })}<\frac{n}{m}\\ \Leftrightarrow & \frac{f(t_{n}^{\prime })}{1-F(t_{n}^{\prime })}<\frac{f(t_{m}^{\prime })}{1-F(t_{m}^{\prime })} \end{array}$$

They also showed in Theorem 1 of [4] that the hazard function is non-increasing on an interval [*x*_1_, *x*_2_] with $t_{0}^{\prime }\in \left[x_{1},x_{2}\right]$. Here $t_{0}^{\prime }$ is the optimal restart time for the algorithm running on a single processor. Since it is known that both $t_{n}^{\prime }>t_{0}^{\prime }$ and $t_{m}^{\prime }>t_{0}^{\prime }$ holds, the desired property follows due to the unimodality of the hazard function.

In other words, if the hazard function is unimodal and the speedup ratio of the parallel computing is sublinear, then the restart times are strictly increasing. This lends evidence that the restriction in Theorem 3.5 to non-decreasing restart times is reasonable in many cases.

Next, we move on and analyse the optimal restart time regarding completion probability. Moorsel and Wolter showed in [8] that in the case of a single processor the optimal restart times are at the equi-hazard intervals, i.e., if *t*_1_, … *t*_*k*_ are optimal restart times, then they fulfil $\frac{f(t_{1})}{1-F(t_{1})}=\cdots =\frac{f(t_{n})}{1-F(t_{n})}$. The completion probability for a single process as in Theorem 3.1 Eq 1 can be stated analogously. This result can be adapted easily to the case of multiple processors. We can express the completion probability of a fixed cut-off algorithm running on multiple processors, as described in Theorem 3.2, equivalently by the following equation.

$$Pr\left(A_{t}^{n}\leq D\right)=1-{\left(1-F\left(t\right)\right)}^{n\cdot k}{\left(1-F\left(t_{0}\right)\right)}^{n}$$ (9)

The extrema of this probability can be attained by equating its derivate to zero. The derivate is given by:

$$-\left(nk{\left(1-F\left(t\right)\right)}^{nk-1}\left(-f\left(t\right)\right){\left(1-F\left(t_{0}\right)\right)}^{n}+nk{\left(1-F\left(t\right)\right)}^{nk}{\left(1-F\left(t_{0}\right)\right)}^{n-1}f\left(t_{0}\right)\right)$$

Equating the derivate to zero yields:

$$\frac{f(t)}{1-F(t)}=\frac{f(t_{0})}{\left(1-F\left(t_{0}\right)\right)}$$ (10)

Since the result by Moorsel and Wolter in [8] and Eq 10 are identical, we can conclude that the optimal restart times (regarding completion probability) are identical. In particular, in case of the fixed cut-off strategy the condition is fulfilled for *D*/*t* = *k*, k∈N.

**Theorem 4.2**. *The optimal restart time regarding completion probability for a fixed-cutoff strategy running on a single processor is identical to the optimal restart time for a fixed-cutoff strategy running on multiple processors*.

Another implication of Lemma 4.1 together with Theorem 4.2 should be pointed out. When considering a parallel fixed cut-off algorithm such that the underlying hazard function is unimodal, the optimal restart time regarding expected runtime and the optimal restart time regarding completion probability are not identical.

## 5 Discussion

In this section, we analyse the results of this work. Shylo et al. used the speedup ratio to measure the effect of parallelisation on the expected runtime in [4]. For measuring the ‘speedup’ in the case of probabilities we choose to use the odds ratio which we briefly introduce at this point.

There are two common ways to describe the likelihood of an event: probabilities and odds. On the one hand, probabilities describe the chance of an event happening compared to all possible outcomes. On the other hand, odds describe the likelihood of an event compared to its complementary event. For example, if the odds of an event are 10 then the event is 10 times as likely as its complementary event. Both, probabilities and odds can be easily transformed into each other.

**Definition 5.1** ([10]). *Let A be an event*. *Then the odds R(A) of an event are defined by:*

$$R\left(A\right)=\frac{Pr(A)}{1-Pr(A)}.$$ (11)

*Conversely*, *given the odds R(A) of an event*, *the probability can be obtained by:*

$$Pr\left(A\right)=\frac{R(A)}{R(A)+1}.$$ (12)

With this definition the odds value can be used as a measurement of certainty. High values indicate, in our case here, that it is very likely to find a solution within the deadline, while low values indicate the inverse. While this is already a good metric for our purposes, we also want to provide a different explanation on how odds can be used as a metric for the quality of a randomised algorithm with an imposed deadline. Let *A* be such an algorithm and *D* be the used deadline. Assume that 0 < *p* < 1 is the probability that *A* finds a solution within the deadline *D*, and *A*_*e*_ is the event of *A* finding a solution within the deadline. We define a new restart algorithm *B* based on *A*’s behaviour. First start *A*, if *A* did not find a solution within *D* steps, restart *A*. Repeat this scheme until a solution is found. Let *X* be a random variable which counts the number of failed runs of *A* and observe its expected value:

$$E\left[X\right]=\sum_{i=1}^{\infty }Pr\left(X\geq i\right)=\sum_{i=1}^{\infty }{\left(1-p\right)}^{i}=\frac{1}{p}-1=\frac{1-p}{p}=R{\left(A_{e}\right)}^{-1}$$

An increase in the odds value can be measured by the odds ratio. Which is defined as follows

**Definition 5.2** ([10]). *Let* *A*_1_, *A*_2_ *be events with* *Pr*(*A*_1_) = *p*_1_ *and* *Pr*(*A*_2_) = *p*_2_. *Then the odds ratio with respect to* *A*_1_ *and* *A*_2_ *is defined by:*

$$OR\left(A_{1},A_{2}\right)=\frac{p_{1}\cdot \left(1-p_{2}\right)}{p_{2}\cdot \left(1-p_{1}\right)}$$ (13)

*The logarithmic scaled odds ratio is given by:*

$$\log OR\left(A_{1},A_{2}\right)=\log\left(p_{1}\cdot \left(1-p_{2}\right)\right)-\log\left(p_{2}\cdot \left(1-p_{2}\right)\right)$$ (14)

Therefore, if the odds ratio is greater than one, the expected number of restarts, as described above, is lower than in the original algorithm. In the following, we first provide empirical evidence that the odds ratio scales linearly with the number of processors if the restart times are identical. Then we show this result theoretically.

**Fig 1:**
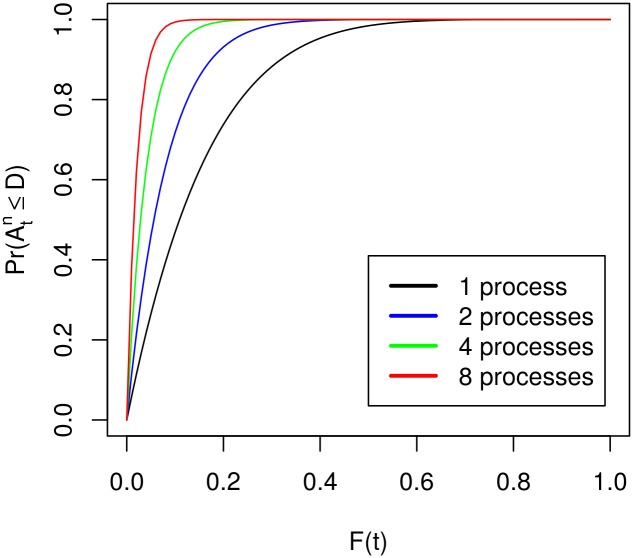
Probabilities with identical restart times. Displayed for 6 restarts.

In the following evaluation all logarithms are natural logarithms. Due to its simplicity, we start by analysing Theorem 3.4. Fig 1 shows the chances of completion for 6 restarts. It is easy to see, that for an increasing number of processors the probability of completing within the deadline converges to 1 much quicker. For a larger number of restarts all of the curves converge to 1 faster, the general statement, however, remains the same. Indeed, the data from experiments matches the theoretical results neatly. We chose to use a SAT solver to examine the results and used problems from the SATLIB [11] to examine the results. The results can be found in the supporting information. In S1 and S2 Tables the completion probabilities are observed. The average number of restarts which correlates to the upper bound of the expected runtime from Theorem 3.2 is exmined in S1 Fig.

**Fig 2:**
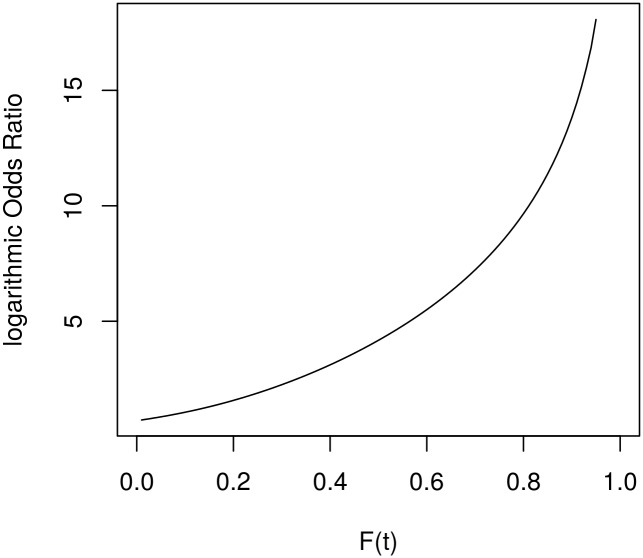
OR Diagram comparing 1 and 2 processors. Using 6 restarts.

Fig 2 shows the log-scaled odds ratio comparing the probabilities of a restart strategy running on two processors to a restart strategy running on one processor. Again 6 restarts were used to plot this graph. For increasing values of *F*(*t*) the log-scaled odds growth appears to be superlinear, possibly even exponential. For more than 6 restarts the odds ratio increases even faster.

**Fig 3:**
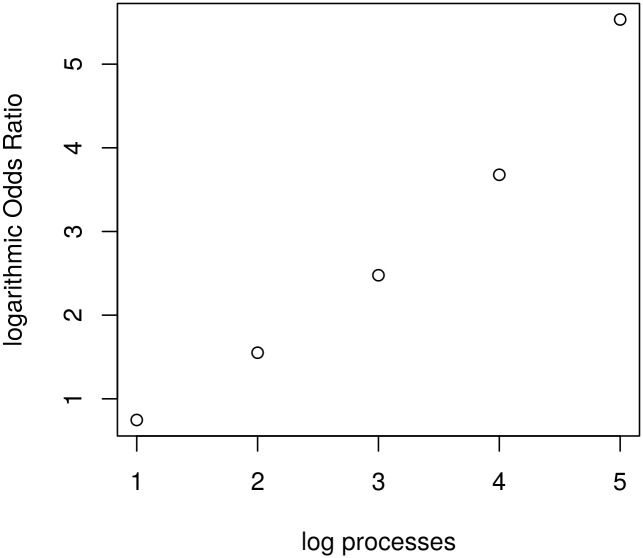
OR Diagram for an increasing number of processors. For (1 − *F*(*t*))^*k*^ = 0.75.

Fig 3 represents the log-scaled odds ratio of 2 to 2^5^ processors compared to a single process. The number of restarts and the probability are chosen such, that (1 − *F*(*t*))^*k*^ = 0.75 holds. Again, the data appears to suggest a superlinear increase in the log-scaled odds ratio. However, it is dependent on the values which were chosen for *F*(*t*) and *k*. If, for example, (1 − *F*(*t*))^*k*^ is set to 0.9, then the log-scaled odds ratio for the case of 2^5^ processors is about 5.53. Increasing the number of processors to 2^8^ yields a log-scaled odds ratio of about 29.17. For small values of (1 − *F*(*t*))^*k*^ the log-scaled odds ratio increases much faster. Indeed, we show that the odds ratio increases superlinearly regardless of the value of *F*(*t*).

**Theorem 5.3**. *Let* 0 ≠ *F*(*t*) ≠ 1, $p_{1}=Pr\left(A_{t}^{n}\leq D\right)$ *with* *n* = 2^*i*^ *for any* i>0,i∈N *and* *p*_2_ = *Pr*(*A*_*t*_ ≤ *D*), *then* $OR\left(\left(A_{t}^{n}\leq D\right),\left(A_{t}\leq D\right)\right)\in \omega \left(n\right)$.

*Proof*. We start by examining the odds ratio.

$$\begin{array}{l} \frac{Pr(A_{t}^{n}\leq D)(1-Pr(A_{t}\leq D))}{Pr(A_{t}\leq D)(1-Pr(A_{t}^{n}\leq D))}\\ =\frac{Pr(A_{t}\leq D)\prod_{j=0}^{i-1}(1+{\left(1-F(t)\right)}^{k2^{j}}{\left(1-F(t_{0})\right)}^{2^{j}}){\left(1-F(t)\right)}^{k}(1-F(t_{0}))}{Pr(A_{t}\leq D){\left(1-F(t)\right)}^{k2^{i}}{\left(1-F(t_{0})\right)}^{2^{i}}}\\ =\frac{\prod_{j=0}^{i-1}(1+{\left(1-F(t)\right)}^{k2^{j}}{\left(1-F(t_{0})\right)}^{2^{j}})}{{\left(1-F(t)\right)}^{k(2^{i}-1)}{\left(1-F(t_{0})\right)}^{2^{i}-1}}\geq \frac{1}{{\left(1-F(t)\right)}^{k(2^{i}-1)}{\left(1-F(t_{0})\right)}^{2^{i}-1}} \end{array}$$

It is already clear that this term is approaching infinity for *i* → ∞. Now we consider the threshold value compared with 2^i^.

$$\lim_{i\to \infty }\frac{\frac{1}{{\left(1-F\left(t\right)\right)}^{k(2^{i}-1)}{\left(1-F\left(t_{0}\right)\right)}^{2^{i}-1}}}{2^{i}}$$

Since both terms approach infinity we can apply L’Hospital’s rule to examine the terms separately. We consider the derivate of the numerator first.

$$-\frac{\ln\left(2\right)\left[k\ln\left(1-F\left(t\right)\right)+\ln\left(1-F\left(t_{0}\right)\right)\right]2^{i}}{{\left(1-F\left(t\right)\right)}^{k(2^{i}-1)}{\left(1-F\left(t_{0}\right)\right)}^{2^{i}-1}}$$

On the other hand $\frac{d2^{i}}{di}=ln\left(2\right)2^{i}$. Therefore we have:

$$\lim_{i\to \infty }-\frac{k\ln\left(1-F\left(t\right)\right)+\ln\left(1-F\left(t_{0}\right)\right)}{{\left(1-F\left(t\right)\right)}^{k(2^{i}-1)}{\left(1-F\left(t_{0}\right)\right)}^{2^{i}-1}}=\infty$$

This shows the claim.

Finally, we consider Theorem 3.5. Some additional assumptions have to be made to analyse the result. Since the restart times are non-decreasing, for $n_{i}=2^{i}\cdot k_{0}^{i}$ the value for *F*(*t*_*j*_) has to be evaluated separately for every 0 ≤ *j* ≤ *i*. We used a bounded growth function to model these circumstances. The used function can be described as: *F*(*t*_*j* + 1_) = *F*(*t*_*j*_) + 0.01 ⋅ (1 − *F*(*t*_*j*_)). Here, we are only portraying the lower bound of Theorem 3.5.

**Fig 4:**
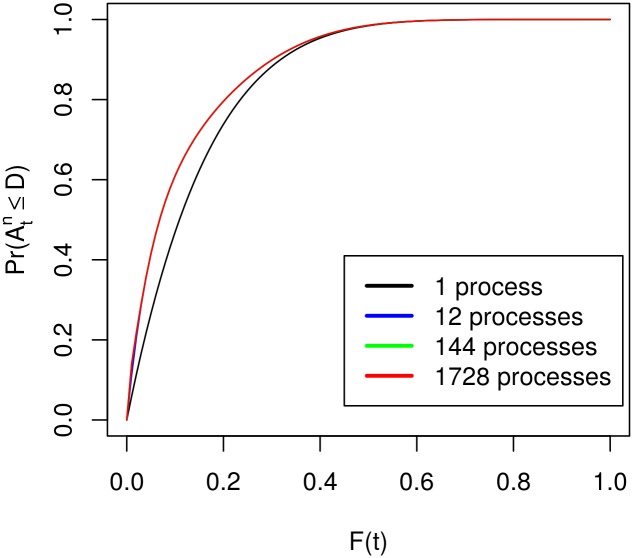
Probabilities in the case of unequal restart times. For 6 restarts.

Fig 4 displays the probability of a single process compared to the probability of multiple processors. It is easy to see that the effect of parallelisation is not as big as in the previous case. In fact, the probabilities of 12, 144 and 1728 processors are notably different only for very low values of *F*(*t*). As before this graph was plotted for the case of 6 restarts. If more restarts are allowed all methods converge faster towards 1 and the area of notably different probabilities shifts more towards *F*(*t*) = 0.

**Fig 5:**
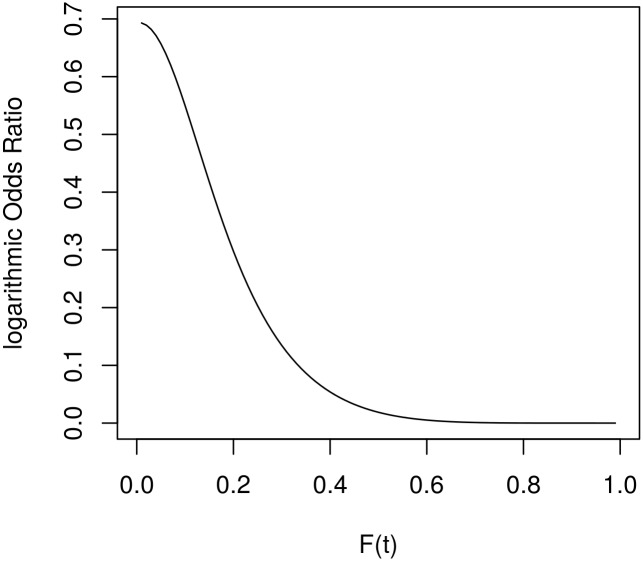
Diagram comparing 1 and 12 processors. For 6 restarts.

Fig 5 shows the log-scaled odds ratio comparing 1 and 12 processors for the case of 6 restarts. While the result of Theorem 3.4 implies an unbounded growth, this result shows, that the OR diagram already starts at its maximum and then decreases monotonically. Two effects can be observed for a greater number of restarts. Firstly the log-scaled OR diagram converges much faster towards 0, secondly, the maximum of the plot decreases.

**Fig 6:**
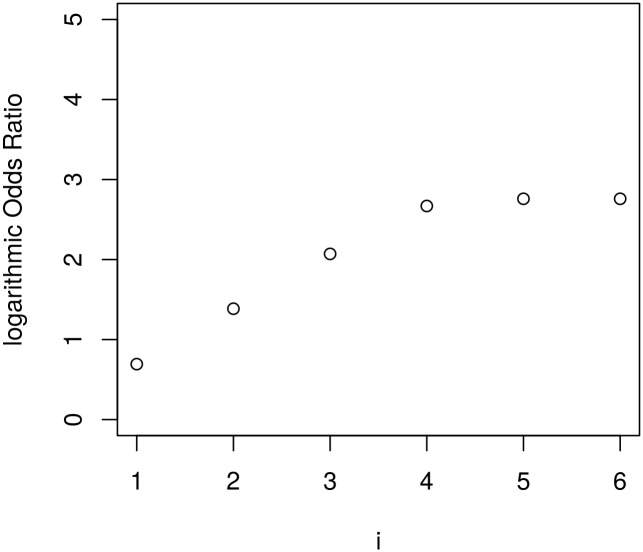
OR Diagram for 2^*i*^ ⋅ 6^*i*^ processors. Using *F*(*t*) = 0.00001.

Finally, we are analysing the log-scaled odds ratio for an increasing number of processors. Fig 6 shows this for 6 restarts and up to 2^6^ ⋅ 6^6^ processors for the case of *F*(*t*) = 0.00001. For the first few steps the log-scaled odds ratio increases with about linear speed but then stagnates. The stagnating behaviour is reached earlier for both: An increased number of restarts or a higher probability *F*(*t*).

## 6 Conclusion

Throughout this work, we have shown several interpretations of the completion probability of a restarted algorithm operating on several processors. In Theorem 3.4 we have provided an explicit way to compare the completion probability of a restarted algorithm running on a single core to the same algorithm running on multiple cores in parallel. Later we analysed this result for several values of *F*(*t*) and provided evidence that under the assumptions of this theorem the probability scales very well with the number of processors. Regarding the odds ratio it even scales superlinearly, as was shown in Theorem 5.3.

However, it is known that the optimal restart (regarding the expected runtime) in the case of a single processor and in the case of multiple processors are possibly not equal. Therefore, the result of Theorem 3.5 is of special interest. Here we operated under the assumptions that the restart times for an increasing number of processors are non-decreasing. Later in the analysis, we provided evidence that for the lower bound a notable difference can only be achieved for low values of *F*(*t*) and the result scales badly for an increasing amount of processors. Of course, it is possible that the actual values are much better than the ones presented here.

We then examined two different notions of optimal restart times. First we showed in Lemma 4.1 that for an unimodal hazard function the restart times are strictly increasing for an increasing number of processors. This lends significance to Theorem 3.5 which is applicable when the algorithm is optimised regarding its expected runtime. On the other hand, we showed in Theorem 4.2 that the optimal restart times regarding completion probability are equal for all number of processors, therefore in this case Theorem 3.4 can be applied. However, it should be noted that finding the optimal restart time requires knowledge about the underlying runtime distribution which can vary vastly depending on the input. Having access to the runtime distribution is often an unnatural assumption in real applications. Therefore, it may be necessary to use non-optimal values as restart times and non-increasing values for the parallel case. Neither the result in Theorem 3.4 nor in Theorem 3.5 requires optimal restart times which makes all of those results interesting for real applications.

## Supporting Information

S1 Table Solved instances of the SAT problem. The used instance was “uf250-04.cnf” from the SATLIB library [11].
(PDF)

S2 Table Probabilities of the experiments. Showing the relative probabilities for a single processor, four processors and the projected probability by using the single relative probability.
(PDF)

S1 Fig Experimental data on the number of restarts. The used instance was “uf250-04.cnf” from the SATLIB library [11]. The experiments were run on four processors in parallel. The depicted upper bound correlates with Theorem 3.2. The data from this experiment differs from the previous data since the experiment had to be redesigned to measure the expected number of restarts.
(TIFF)

S1 File Experimental Data depicted in S1 Table. The file “S1_File.zip” is the data gathered from the experiments regarding completion probability.
(ZIP)

S2 File Experimental Data depicted in S1 Fig. The file “S2_File.zip” is the data gathered from the experiments regarding expected runtime.
(ZIP)
