# Supplementary material for: Completion Probabilities and Parallel Restart Strategies under an Imposed Deadline
Source: PLoS One. 2016 Oct 12;11(10):e0164605. doi: 10.1371/journal.pone.0164605 (PMC5061357; doi:10.1371/journal.pone.0164605)
Supplement: S1 Table — The used instance was “uf250-04.cnf” from the SATLIB library [11]. (PDF) [file pone.0164605.s001.pdf]

Deadline	Fulfilled single	Single not fulfilled	Parallel fulfilled	Parallel not fulfilled
100.8	91	2909	363	2637
350.144	97	903	318	682
400.2	347	2653	1174	1826
450.244	123	877	390	610
550.344	163	837	507	493
650.444	186	814	513	487
750.544	204	796	576	424
850.644	216	784	638	362
950.744	221	779	669	331
1050.844	259	741	719	281
1150.944	279	721	751	249
1251.044	323	677	802	198
1350.144	327	673	820	180
1450.244	388	612	826	174
1550.344	396	604	844	156
1650.444	398	602	864	136
1750.544	415	585	880	120
1850.644	397	603	885	115
1950.744	448	552	894	106
2050.844	480	520	922	78
2150.944	493	507	928	72
2251.044	484	516	929	71
2310.144	5054	4946	9391	609
2350.144	516	484	957	43
2450.244	536	464	939	61
2550.344	553	447	964	36
2650.444	542	458	959	41
2750.544	530	470	963	37
2850.644	582	418	973	27
2950.744	594	406	972	28
3050.844	583	417	971	29
3150.944	599	401	979	21
3251.044	634	366	974	26
3350.144	614	386	985	15
3450.244	631	369	984	16
3550.344	661	339	984	16
3650.444	644	356	988	12
3750.544	688	312	982	18
3850.644	658	342	986	14

3950.744	694	306	996	4
4050.844	713	287	990	10
4150.944	711	289	992	8
4251.044	743	257	989	11
4350.144	703	297	992	8
4450.244	741	259	996	4
4550.344	751	249	996	4
4620.288	7467	2533	9961	39
4650.444	736	264	995	5
4750.544	775	225	995	5
4850.644	766	234	998	2
4950.744	783	217	996	4
5050.844	786	214	995	5
5150.944	784	216	998	2
5251.044	778	222	998	2
7674.132	9049	951	10000	0
9984.276	9525	475	10000	0
