# Supplementary material for: Completion Probabilities and Parallel Restart Strategies under an Imposed Deadline
Source: PLoS One. 2016 Oct 12;11(10):e0164605. doi: 10.1371/journal.pone.0164605 (PMC5061357; doi:10.1371/journal.pone.0164605)
Supplement: S2 Table — Showing the relative probabilities for a single processor, four processors and the projected probability by using the single relative probability. (PDF) [file pone.0164605.s002.pdf]

Deadline	Relative probability single	Relative probability parallel	Projected probability
100.8	0.03033	0.121	0.1159234602
350.144	0.097	0.318	0.3351081627
400.2	0.11566	0.39133	0.3884049228
450.244	0.123	0.39	0.4084405814
550.344	0.163	0.507	0.5092030762
650.444	0.186	0.513	0.5609665408
750.544	0.204	0.576	0.5985307645
850.644	0.216	0.638	0.6221980017
950.744	0.221	0.669	0.6317440007
1050.844	0.259	0.719	0.6985100554
1150.944	0.279	0.751	0.7297653347
1251.044	0.323	0.802	0.7899345278
1350.144	0.327	0.82	0.794855321
1450.244	0.388	0.826	0.8597167921
1550.344	0.396	0.844	0.8669092861
1650.444	0.398	0.864	0.8686633408
1750.544	0.415	0.88	0.8828820494
1850.644	0.397	0.885	0.8677884951
1950.744	0.448	0.894	0.9071554724
2050.844	0.48	0.922	0.92688384
2150.944	0.493	0.928	0.9339258116
2251.044	0.484	0.929	0.9291077425
2310.144	0.5054	0.9391	0.9401565741
2350.144	0.516	0.957	0.9451241265
2450.244	0.536	0.939	0.9536476324
2550.344	0.553	0.964	0.9600763635
2650.444	0.542	0.959	0.9559990643
2750.544	0.53	0.963	0.95120319
2850.644	0.582	0.973	0.9694715238
2950.744	0.594	0.972	0.9728290931
3050.844	0.583	0.971	0.9697626157
3150.944	0.599	0.979	0.9741430384
3251.044	0.634	0.974	0.9820557901
3350.144	0.614	0.985	0.977800192
3450.244	0.631	0.984	0.9814601821
3550.344	0.661	0.984	0.9867931638
3650.444	0.644	0.988	0.9839379863
3750.544	0.688	0.982	0.9905241457
3850.644	0.658	0.986	0.9863194227

3950.744	0.694	0.996	0.9912322995
4050.844	0.713	0.99	0.9932153478
4150.944	0.711	0.992	0.9930242426
4251.044	0.743	0.989	0.9956375296
4350.144	0.703	0.992	0.9922191723
4450.244	0.741	0.996	0.9955001394
4550.344	0.751	0.996	0.996155876
4620.288	0.7467	0.9961	0.9958833802
4650.444	0.736	0.995	0.9951424676
4750.544	0.775	0.995	0.9974371094
4850.644	0.766	0.998	0.9970017805
4950.744	0.783	0.996	0.9977826261
5050.844	0.786	0.995	0.9979027264
5150.944	0.784	0.998	0.9978232177
5251.044	0.778	0.998	0.9975710873
7674.132	0.9049	1	0.9999182059
9984.276	0.9525	1	0.9999949093
