# Supplementary figures and images for: Completion Probabilities and Parallel Restart Strategies under an Imposed Deadline
Source: PLoS One. 2016 Oct 12;11(10):e0164605. doi: 10.1371/journal.pone.0164605 (PMC5061357; doi:10.1371/journal.pone.0164605)

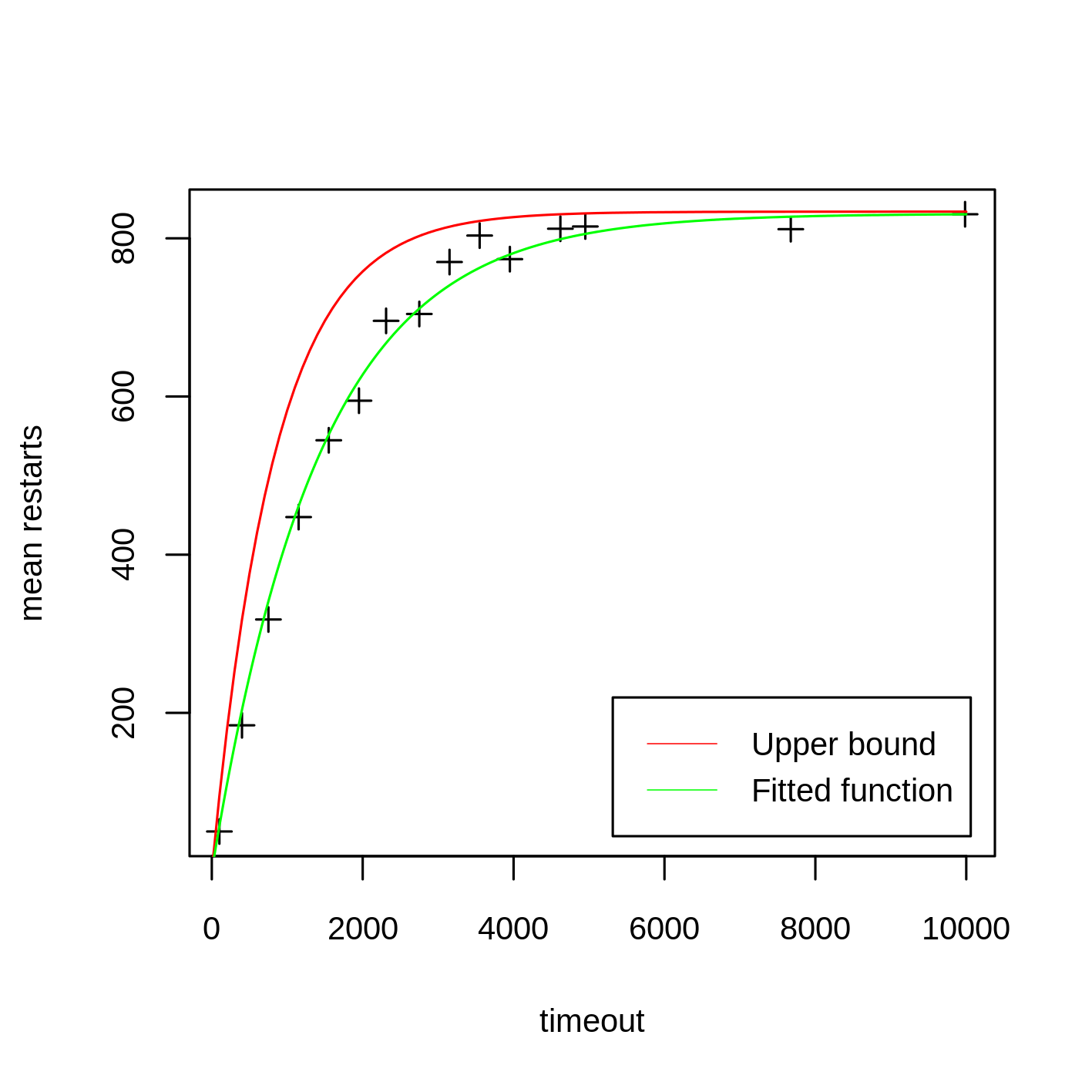

Supplement: S1 Fig — The used instance was “uf250-04.cnf” from the SATLIB library [11]. The experiments were run on four processors in parallel. The depicted upper bound correlates with Theorem 3.2. The data from this experiment differs from the previous data since the experiment had to be redesigned to measure the expected number of restarts. (TIFF) [file pone.0164605.s003.tiff]
